# The potential therapeutic effects of *Lactobacillus plantarum* and inulin on serum and testicular reproductive markers in diabetic male rats

**DOI:** 10.1186/s13098-020-00560-0

**Published:** 2020-06-26

**Authors:** Marziyeh Rahimiyan-Heravan, Leila Roshangar, Pouran Karimi, Safa Sefidgari-Abrasi, Mohammad Morshedi, Maryam Saghafi-Asl, Khadijeh Bavafa-Valenlia

**Affiliations:** 1 Student Research Committee, Tabriz University of Medical Sciences, Tabriz, Iran; 2 Drug Applied Research Center, Tabriz University of Medical Sciences, Tabriz, Iran; 3 Stem Cell Research Center, Tabriz University of Medical Sciences, Tabriz, Iran; 4 Neurosciences Research Center, Tabriz University of Medical Sciences, Tabriz, Iran; 5 Nutrition Research Center, School of Nutrition and Food Sciences, Tabriz University of Medical Sciences, Tabriz, Iran; 6 Department of Clinical Nutrition, School of Nutrition and Food Sciences, Tabriz University of Medical Science, Tabriz, Iran

**Keywords:** Diabetes mellitus, Infertility, Lactobacillus *plantarum*, Inulin, Kisspeptin

## Abstract

**Background:**

It is well established that diminished reproductive health is one of the notable long-term outcomes of type 2 diabetes mellitus (T2DM), especially among males. Due to the global increasing rate of T2DM and infertility, we aimed to investigate the impact of *Lactobacillus plantarum (L. plantarum)*, inulin, and their combinatory supplementation on fertility markers as well as testicular kisspeptin and androgen receptor (AR)’s expression in diabetic male rats.

**Methods:**

Thirty-five Male Wistar rats with Streptozotocin-induced T2DM were supplemented with *L. plantarum*, inulin, or their combination for 8 weeks. At the end-point, the animals were sacrificed and serum, testicular, and seminal parameters were studied.

**Results:**

Administration of *L. plantarum* and inulin in diabetic male rats improved sperm motility and viability (P < 0.001, both) as well as testicular tissue development via increasing leydig cell number, testicular spermatid count, and diameter of seminiferous tubules (P < 0.001, all). Testicular expression of Kisspeptin was elevated by inulin supplementation (P = 0.01). *L. plantarum* administration increased testicular AR expression (P = 0.01). The expression of Kisspeptin showed a remarkable correlation with fertility markers (P < 0.001).

**Conclusion:**

Supplementation with either *L. plantarum*, inulin, or their combination can prevent infertility caused by T2DM in male rats via improving testicular kisspeptin and AR expression, leydig cell count, and effectively increasing epididymal sperm motility and viability.

## Background

Diabetes is one of the commonly diagnosed metabolic diseases around the world. The prevalence of diabetes is expected to triple in Africa and the Middle East and to double in America and Europe by 2025. Approximately, 422 million adults over 18 years old are suffering from Type 2 Diabetes Mellitus (T2DM), based on the WHO’s statistics in 2014 [49]. T2DM leads to different micro- and macro-vascular complications which are being vastly investigated; yet its effects on infertility are not completely declared.

On average, 40 percent of couples’ fertility problems are due to male hypogonadism. It is suggested that 51 percent of cases with T2DM are facing subfertility [23]. After adjustment for age and BMI, the rate of hypogonadism among men with diabetes types 1 and 2 is higher than healthy ones [7].

Fertility is the consequence of accurate function of the Hypothalamus-Pituitary-Gonad (HPG) axis, majorly mediated by kisspeptin, the main GnRH stimulator in the hypothalamus [15]. It was recently found that kisspeptin is translated and produced in several other organs like testis which could be pivotal for its development; however, the exact role is still uncertain [9, 19, 40].

Several studies have shown that diabetic rats have significantly decreased weight of reproductive organs, sperm count and motility, and lower serum testosterone (T) [1, 50]. This decreased male reproductive ability as a result of T2DM could be linked to hyperinsulinemia and its inhibitory effect on normal spermatogenesis or decreasing the production of sex hormone binding globulin (SHBG) [13]. In addition, high serum glucose levels could alter normal antioxidant and glycolytic activities in Sertoli cells (SC) of the testis by blocking interleukine-10 (IL-10) and subsequently, improving sperm DNA damage [13, 44].

Animal studies have revealed that the gut microbiota plays a significant role in the development of diabetes and its possible complications [45, 46]. Probiotics are the living microorganisms with beneficial effects on host health condition [18]. But prebiotics are kind of indigestible food ingredients stimulating growth or activity of the gut microbiota. Mostly known prebiotics include fructooligosaccharides (FOS) and inulin (a class of food fibers called fructans) [21]. Both pro- and prebiotics can increase insulin signaling in T2DM [37]. Synbiotics are the combination of pro- and prebiotics with more useful effects [21]. Probiotics are able to alter carbohydrate metabolism and improve fasting blood sugar (FBS), insulin sensitivity, and oxidative stress in T2DM [37]. On the other hand, studies have shown that the gut microbiota could improve male fertility through producing amino acid metabolites or via being translocated to the organs which affect reproductive system [10]. The gut microbiota-induced butyrate in rats helped testis development as well as improved blood-testis-barrier (BTB) permeability and endocrine function. According to the findings, the gut microbiota and their metabolites play an effective role in semen quality and reproductive health [3].

It was reported that *Lactobacillus rutei* (*L. rutei*) increased the function of seminiferous tubules, spermatogenesis, and the number of leydig cells in male rats [33]. In addition, probiotics in obese and overweight cases improved male fertility via inducing weight loss and decreasing oxidative stress [10]. Lactobacillus *plantarum* (*L. plantarum*) is a probiotic from lactobacillus family which can be found in many food products [11]. It is suggested that *L*. *plantarum* has anti-obesity effects and antioxidant activities [6]. Inulin as well offers many benefits to the host by selectively stimulating the growth and/or activity of intestinal bacteria such as *L. plantarum* [48]. Furthermore, *L. plantarum* utilizes inulin for growth and many previous reports proved that inulin improved the viability of *L. plantarum* [41, 42]. In addition, the probiotic could use inulin as the sole carbon source, suggesting better colonization in the intestine and stimulates *L. plantarum* to produce a higher amount of some bio components, such as butyrate, with beneficial effects for human health [12, 31].

Recent studies interestingly suggested that administration of *L. plantarum* along with inulin promoted serum markers and improved diabetes neurological complications in diabetic male rats [29, 46]. In addition *L. plantarum* in combination with inulin showed antioxidative and anti-apoptotic effects in gut and heart, respectively [20, 39]. A significant increase of SOD, GPx, and catalase(CAT) as well was observed after concurrent administration of Lactobacillus casei and inulin among healthy volunteers Kleniewska et al. [26].

To the best of our knowledge, studies on the effects of *L. plantarum* or inulin on male fertility are rare to none. Regarding the increasing prevalence of T2DM and the resultant male fertility problems and previously proved anti-oxidative and anti-apoptotic impacts of *L. plantarum* and inulin, this experimental study was aimed to investigate the effects of separate and concurrent supplementation of *L. plantarum* as well as inulin on testicular function via analyzing serum, tissue, and semen markers.

## Materials and methods

### Animals

All the experimental protocol was approved by the Institutional Animal Ethics Committee (IR.TBZMED.REC.1395.1239) and conformed to the Iranian National Science Academy Guidelines for the use and care of experimental animals in this research. Thirty-five male Wistar rats (aged 6 ± 1 weeks) were purchased from Tabriz University of Medical Sciences (TBZM), Tabriz, Iran. Their average weight was 200 ± 20 grams. The animals were kept in standard temperature of 22–25 °C, artificial 12 h–12 h light–dark cycle, and 40–60% of humidity inside standard separate cages per 4 rats. Animal access to water and food sources was ad libitum. Primarily, all rats were fed a normal pellet diet (NPD: 12% fat, 22% protein, and 66% carbohydrate) for 1 week with the aim of adaptation to the new environment. The daily amount of food consumed as well as weekly body weight were recorded. Later, the animals were randomly allocated into two groups: the NPD-fed group or the high-fat diet (HFD: 58% fat, 17% protein, and 25% carbohydrate) (Table 1) fed group, consuming for 4 weeks.

**Table 1:** Composition of the high-fat diet (HFD)

Composition	Percent
Powdered NPD	42
Cholesterol	1
Ghee	25
Sucrose	15
Flour	15
Cholic acid	0.5
Pea flour	0.5

### T2DM induction

After 12 h of fasting (from 19:00 to 07:00), the HFD groups were injected by a single dose of STZ ((35 mg/kg in 50 μL citrate buffer (0.1 M, pH: 4.5), intraperitoneal (IP). After 72 h, blood samples were obtained from the tail of the rats. Fasting blood glucose (FBG) ≥ 250 mg/dL was considered as diabetic. The control group was kept in a standard condition with no STZ. All the rats were fed the NPD, until the end of the study. Overall, the rats were allocated into six following groups: 1] Diabetic + *L. plantarum* (DL, n = 6); 2] Diabetic + inulin (DI, n = 6); 3] Diabetic + *L. plantarum* + inulin (DLI, n = 6); 4] Diabetic Control (DC, n = 6); 5] Diabetic Sham (Dsh, n = 5); and, 6] Normal Control (HC, n = 6).

### Supplementation

*Lactobacillus plantarum* ATCC 8014 was purchased from Biotechnology Research Center, TBZMED, Iran. The process of culturing the bacteria is described elsewhere [29]. *L. plantarum* was mixed with phosphate-buffer-sodium (PBS) at the concentration of 10^7^ colony-forming units per milliliter (CFU/mL) and kept inside the refrigerator. The amount of inulin, dissolved in water, was calculated as 5% of food weight consumed daily. The weight of the rats was weekly recorded. Gastric gavage was done every 24 h for each rat. The supplementation continued for 8 weeks. The DSh rats were gavaged with saline in order to induce gavage stress.

### Blood and tissue samples

At the end-point, after a12-h fasting, the animals were anesthetized with sodium pentobarbital (65 mg/kg BW IP, Sigma). Then, 4 mL of blood was immediately obtained from the heart of the rats and moved into the tubes. Blood samples were centrifuged at 10,000 rpm at 4 °C for 20 min. The separated sera were obtained and kept in the freezer at − 80 °C, until biochemical assays.

All the animals were rapidly decapitated. The abdominal cavity was opened and male reproductive organs, including both testis were removed and weighed to later determine gonadosomatic index. This index is the ratio of paired testis weight to total body weight. The left testis was selected for conducting further analyses. The testis was longitudinally cut in half, using a sharp blade. One half was floated inside a fixative (i.e. 10% neutral buffered formalin (3.7% formaldehyde in H_2_O)) for further assays. The other half was cut in two once more. One was homogenized with PBS (0.5 cc PBS per 0.1 g tissue) and centrifuged for 10 min at 9000 rpm at 4 °C. The other aliquot was kept in the freezer at − 80 °C, until use.

### Western Blotting

Western blotting technique was applied in order to evaluate the expression of testicular kisspeptin and androgen receptor. Primarily, the proteins of 10 µL of the samples were separated by electrophoresis in a 10% acrylamide gel, containing SDS-PAGE. Proteins were transferred to polyvinylidene difluoride (PVDF) membranes (Sigma), using a transfer buffer and a Bio-Rad Scientific Instruments Transphor Unit. PVDF membranes were incubated with primary antibodies (Santa Crus) overnight at 4 °C; followed by incubation with anti-rabbit or anti-mouse secondary antibodies (Santa Crus). Pictures were then taken with a Fujifilm LAS-3000 in order to quantify the immunoreactive proteins (Fujifilm, Tokyo, Japan). Density of bonds was determined using Image J software (National Institute of Health, Bethesda, Maryland, USA). As an internal control, the immunoblots were probed with an antibody recognizing β-Actin, prepared by the same procedure.

### Biochemical analysis

Serum and tissue testosterone concentrations were detected, using enzyme-linked immunoassay (ELISA) kits (Bioassay Technology Laboratory, Shanghai Korain Biotech Co. China. E0930Ra1), following manufacturer’s instructions. Intra- and inter-assay CVs were < 8% and < 10%, respectively.

### Testis histomorphometric analysis

In order to perform histomorphometric examinations, the fixed tissues were terminated and embedded in paraffin and then, cut into sections (4–6 μm thickness). After preparation, Haematoxylin and eosin staining (H & E stain) was performed. Light microscope was used for counting the cells and capturing images (×40) from each sample. The tissues were analyzed morphometrically, using Image J software.

### Epididymal sperm analysis

The bilateral caudal epididymis was dissected out and spermatozoa were collected in 2 mL medium (Hams F_10_), containing 0.5% bovine serum albumin. After 5 min of incubation at 37 °C, sperm count as well as progressive, non-progressive, and immotile sperms were determined, using the standard hemocytometric method, according to WHO laboratory manual protocol for the examination of human semen (5^th^ Edition) (2010) [32].

### Statistical analysis

Data were presented as mean ± SD for each treatment group. SPSS software (version 25) was applied for statistical analysis as well as drawing charts and graphs. One-way analysis of variance (ANOVA), followed by Tukey’s test as post hoc analysis, was utilized to examine the level of significance between groups. Correlations between two variables were carried out, using the Pearson correlation coefficient. *P *< *0.05* was regarded as statistically significant.

## Results

### Serum glucose and insulin level and Body Mass Alterations

**Fig. 1:**
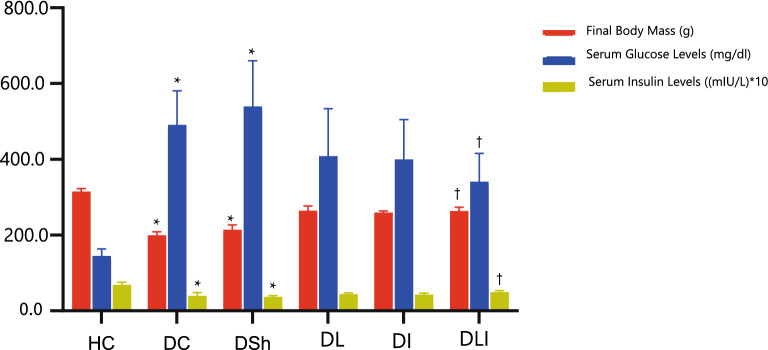
Effects of *L*. *plantarum* and Inulin on final body mass, serum Glucose and Insulin levels. HC, Healthy Control; DC, Diabetic Control; DSh,  Diabetic Sham; DL,  Diabetics treated by *L. plantarum*; DI,  Diabetics treated by inulin; DLI,  Diabetics treated by *L. plantarum* and inulin. Data are presented as mean ± SD, calculated by ANOVA and a post hoc Tukey’s test. Values are regarded as significantly different at P < 0.05. *P < 0.05 compared with the HC group. ^†^P < 0.05 compared with DSh group

Basic results concerning the effects of *L*. *plantarum* and inulin on serum glucose and insulin levels and body mass changes among the study groups are previously reported [29], Valenlia, [29]. While glucose levels in the DL (P < 0.001), DI (P < 0.001), and DLI (P = 0.006) groups were higher than the HC group, the level of serum glucose in the DLI group was significantly lower than DSh group (P = 0.009). DLI as well showed a significant higher insulin levels in comparison to DSh group (P = 0.005). In addition, the treatment groups compared to the control group showed a better weight gain after receiving the supplements (Fig. 1).

### Weight alterations of reproductive organs

**Fig. 2:**
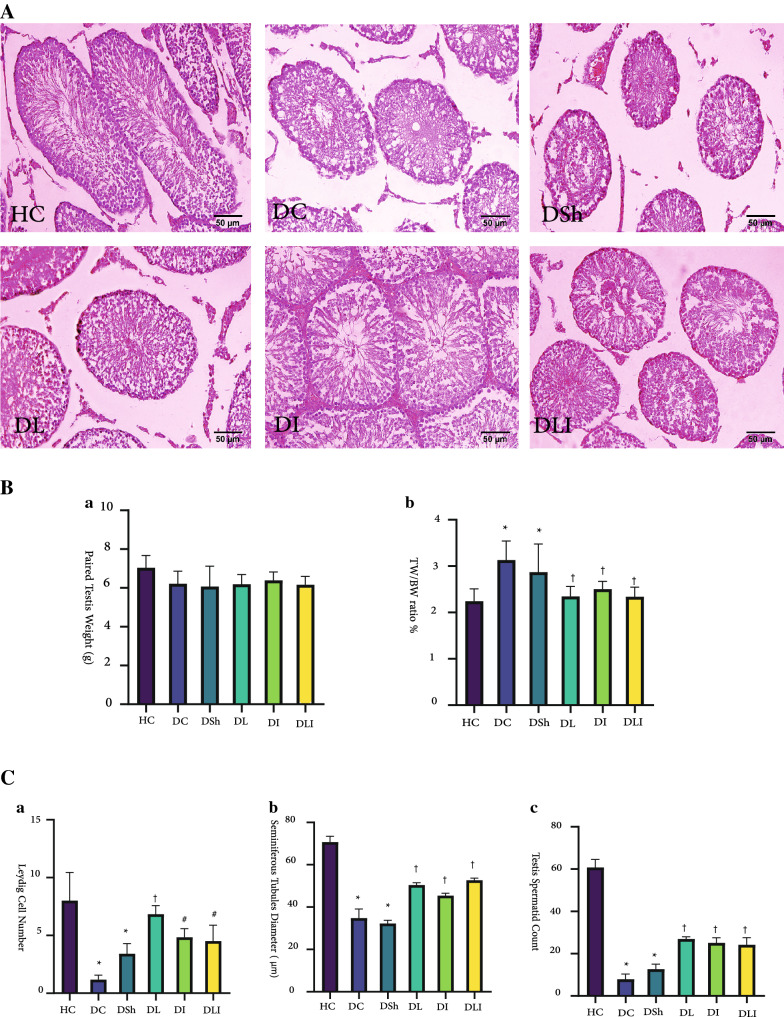
Effects of *L*. *plantarum* and Inulin on testicular parameters. **A** H&E stained sections of Wistar rat s’ testis. **B a, b.** Comparison of final testis weight and TW/BW ratio among groups **C a**, **b.** Relative seminiferous diameter and Leydig cell number. **c** Relative testis spermatid count. HC,  Healthy Control; DC,  Diabetic Control; DSh,  Diabetic Sham; DL,  Diabetics treated by *L. plantarum*; DI,  Diabetics treated by inulin; DLI,  Diabetics treated by *L. plantarum* and inulin. TW/BW,  the ratio of paired testis weight to body weight at the end of the study. Data are presented as mean ± SD and calculated by ANOVA and a post hoc Tukey’s test. Values are regarded as significantly different at P < 0.05. *P < 0.05 compared with the HC group. ^#^P < 0.05 compared with DC group. ^†^P < 0.05 compared with the DSh group

Compared to the HC group, T2DM led to a significantly higher gonadosomatic index (testis/body weight ratio (TW/BW)) in the DC group (P = 0.001), while it could not significantly increase paired testis weight, in comparison to the HC rats (Fig. 2). The 8-week supplementation of *L. plantarum* and inulin in the diabetic groups decreased this ratio to a significant level. The DL, DI, and DLI groups had a lower TW/BW ratio than the DC group (P = 0.002, P = 0.019, and P = 0.002, respectively). Although the index increased in the DSh group, it was not significant, compared to the HC rats (Fig. 2). There was no significant difference among the intervention groups.

### Western blot analysis

#### Testicular expression of Androgen Receptor

**Fig. 3:**
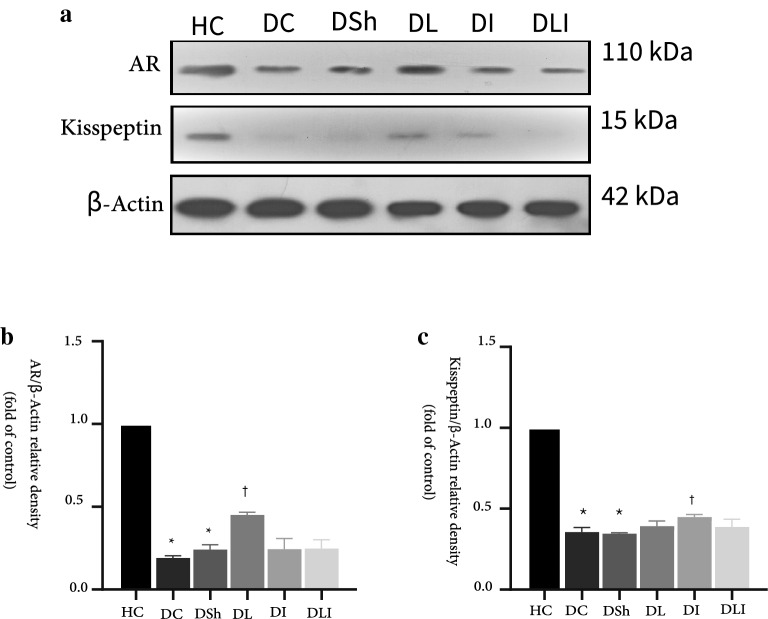
Effects of *L. plantarum* and inulin on Kisspeptin and AR expression in the rat testis. **a** The protein levels of AR in testis from the control and diabetic rats, as determined by western blot analysis. **b** Relative protein quantification of testicular AR, on the basis of β-actin. **c** Relative protein quantification of testicular kisspeptin, on the basis of β-actin. HC,  Healthy Control; DC,  Diabetic Control; DSh,  Diabetic Sham; DL,  Diabetics treated by *L. plantarum*; DI,  Diabetics treated by inulin; DLI,  Diabetics treated by *L. plantarum* and inulin. Data are presented as mean ± SD and calculated by ANOVA and a post hoc Tukey’s test. Values are regarded as significantly different at P < 0.05. *P < 0.05 compared with the HC group. ^#^P < 0.05 compared with the DC group. ^†^P < 0.05 compared with the DSh group

In the present study, diabetes caused a significant decrease in the expression of AR in the DC and DSh groups, compared to the HC rats (P < 0.001). On the other hand, supplemented groups showed an increased AR expression, compared to the DC rats (P = 0.02, for all). However, the DL group had a significantly higher expression level of AR than the DI and DLI rats (P = 0.02) (Fig. 3a). AR expression in testis also showed a strong correlation with testis testosterone level, sperm vitality and tissue parameters (Table 2).

**Table 2:** Correlation coefficients between testicular kisspeptin and AR expression and fertility markers

	Tissue T level	Tissue spermatid count	Leydig cell number	Seminiferous tubule diameter	Sperm vitality
AR/β-actin	0.565 (P = 0.002)	0.931 (P < 0.001)	0.703 (P < 0.001)	0.875 (P < 0.001)	0.582 (P < 0.001)
kisspeptin/β-actin	0.591 (P = 0.001)	0.927 (P < 0.001)	0.608 (P < 0.001)	0.837 (P < 0.001)	0.587 (P < 0.001)

P value is reported based on Pearson correlation test (n = 18). P < 0.05 was regarded as statistically significant

#### Testicular expression of kisspeptin

The results of Western blot analysis showed that diabetes induction caused decreased expression of testicular kisspeptin in both DC and DSh groups, compared to the HC group (P < 0.001). Although supplementation with *L. plantarum* and inulin numerically increased the expression of kisspeptin in the treatment groups, it reached to the significant level only in the DI group (P < 0.001). The kisspeptin expression changes were not significantly different among the intervention groups (Fig. 3b). The kisspeptin expression in testis also showed strong correlation with testis testosterone level, sperm vitality and tissue parameters (Table 2).

### Serum and intra-testicular levels of testosterone

**Fig. 4:**
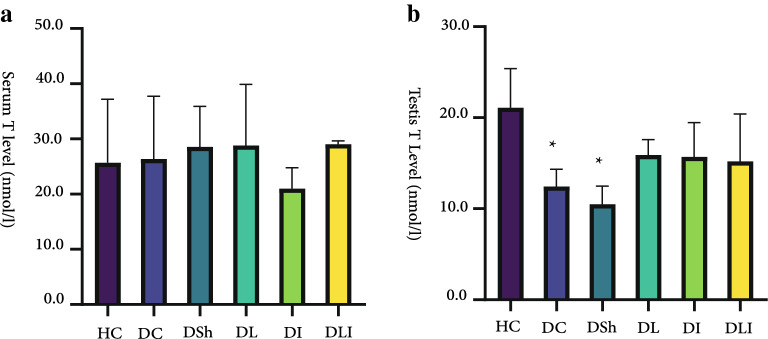
Effects of *L. plantarum* and inulin on serum and tissue values of testosterone (T). **a** in Serum levels of testosterone. **b** Testis levels of testosterone. HC,  Healthy Control; DC,  Diabetic Control; DSh,  Diabetic Sham; DL,  Diabetics treated by *L*. *plantarum*; DI,  Diabetics treated by inulin; DLI,  Diabetics treated by *L. plantarum* and inulin. Data are presented as mean ± SD and calculated by ANOVA and a post hoc Tukey’s test. Values are regarded as significantly different at P < 0.05. * P < 0.05 compared with the HC group

In the present experiment, it was observed that diabetes induction with STZ at the study dosage might not be able to alter serum hormone levels. Concentration of serum testosterone was not significantly different among the study groups (Fig. 4). But tissue analysis indicated different results. We found that intra-testicular levels of testosterone in the DC (P = 0.022) and DSh (P = 0.002) rats was significantly lower than the HC group. In the intervention groups, testosterone level increased numerically, compared to the DC or DSh group, but did not reach the significant level (Fig. 4).

### Histomorphometric findings

Not similar to sperm concentration of epididymal semen, testicular spermatid count showed magnificent changes through the supplementation. While all the rats had a significantly lower spermatid count, compared to the healthy group (P < 0.001, all), the intervention groups showed a higher spermatid number than the DC and DSh (P < 0.001) groups. Therefore, it was predictable that the diameter of seminiferous tubules would increase by *L. plantarum* and inulin supplementation. It was true as both the DC and DSh groups had a significantly lower diameter of seminiferous tubules, compared to the HC group (P < 0.001). The value was higher in all three intervention groups, compared to the DC and DSh rats (P < 0.001). The diameter of seminiferous tubules in the DL (P = 0.011) and DLI (P < 0.001) groups was significantly higher than the DI group. In addition, it was found that the DC and DSh groups had fewer leydig cell numbers, compared to the HC (P < 0.001) group. But the DL and DI groups showed increased leydig cell numbers than the DC rats (P < 0.001). The DLI group also had more leydig cells than the DC rats (P = 0.002). Moreover, the DL group had an increased leydig cell number, compared to the DSh rats (P = 0.002); however, this number was still significantly lower in all the intervention groups than the HC rats. No significant difference was found among the intervention groups (Fig. 2).

### Epididymal sperm density and motility

Compared to the HC group, sperm count in the DSh group showed a decrease of 13 million spermatozoa, though it was not statistically significant. All the DL, DI, and DLI groups had significantly more progressive sperm motility (motility A), compared to the DSh and DC groups (P < 0.001, all). On the other hand, all intervention groups had significantly less immotile sperms (motility C), in comparison to the DSh and DC groups (P < 0.001, for all). The DSh group had a decreased percentage of progressive (P < 0.001) and higher percentage of immotile sperms (P < 0.001) than the HC group. The results of ANOVA showed that sperm viability percents of the DL, DI, and DLI groups were significantly higher than the DSh (P < 0.001) and very similar to the HC group. However, there was no significant difference among the intervention groups. Although non-progressive motility decreased numerically after supplementation, it did not show any significant variation among the intervention groups (Table 3).

**Table 3:** Spermogram Findings

Sperm parameters	HC	DC	DSh	DL	DI	DLI
Sperm concentration (× 10^6^)	41.25 (1.4)	27.7 (1.1)	27.6 (7.1)	32.3 (7.9)	34.2 (6.0)	38.4 (7.7)
Progressive motility (%)	58.3 (14.7)	16.6 (8.1)*	16.0 (5.4)*	53.3(16.4)^#,†^	50.7 (16.3)^#,†^	53.0 (8.9)^#,†^
Non-progressive motility (%)	30.0 (14.1)	31.6 (11.6)	28.0 (8.3)	21.6 (9.8)	20 (8.9)	25.8 (4.9)
Immotile (%)	11.7 (4.0)	51.6 (9.8)*	58.0 (8.3)*	13.3 (5.1)*^,#,†^	16.6 (8.1)^#,†^	14.6 (9.1)^#,†^
Viability (%)	88.3 (4.0)	48.3 (9.8)*	42.0 (8.3)*	86.6 (5.1)^#,†^	83.3 (8.1)^#,†^	82.5(8.7)^#,†^

Data are presented as mean percentage (SD), calculated by ANCOVA and pairwise comparison

Values are regarded as significant at P < 0.05

* P < 0.05, compared with the HC group. ^#^P < 0.05, compared with the DC group. ^†^P < 0.05, compared with the DSh group

HC,  Healthy Control; DC,  Diabetic Control; DSh,  Diabetic Sham; DL,  Diabetics treated by *L. plantarum*; DI,  Diabetics treated by inulin; DLI,  Diabetics treated by *L. plantarum* and inulin. Motility A,  progressive sperms. Motility B,  non-progressive sperms. Motility C,  immotile sperms

## Discussion

To the best of our knowledge, the present study was the first to reveal a significant increase in the number of leydig cells, spermatid count, and the expression of AR via oral supplementation with *L. plantarum* as well as kisspeptin via oral consumption of inulin in the testis of diabetic rats. In addition, we found that the study supplements could improve testicular function, possibly as a result of improved diabetic parameters. The most important to claim is that our results showed a strong positive linear correlation between testicular kisspeptin expression and fertility markers. This finding is an affirmation to the previous studies that tried to respond to this challenge about whether testicular kisspeptin expression can impact markers of male reproduction.

As shown earlier, diabetes causes weight loss of body and reproductive organs due to decreased circulating leptin concentration and inducing oxidative stress and testicular atrophy, respectively [17, 27, 36]. Yongde et al. [50] suggested that diabetes-induced rats by STZ injection had decreased testis weight, compared to healthy controls. Based on our data, gonadosomatic index of the rats was significantly improved after feeding with the supplements. Earlier, Bavafa et al. [46] found that *L. plantarum* and inulin supplementation improved polyphagia and weight loss along with reducing FBS levels in diabetic male rats. It was suggested that the hypothalamic insulin and leptin could prevent polyphagia through reduced appetite and food intake by stimulating the proopiomelanocortin (POMC) neurons [30]. Bavafa et al. (Valenlia, [29] showed that *L. plantarum* and inulin administration could increase the hypothalamic insulin and leptin levels and decrease food intake, compared to diabetic controls. In addition, in our else previous work, we showed that *L. plantarum* and inulin supplementation ameliorated oxidative stress status [29]. Regarding later findings, preserved body weight as a result of developed diabetic status as well as preserved/increased testis weight because of decreased oxidative stress could subsequently lead to an increased gonadosomatic index within supplemented groups. This relation was verified according to the positive correlation between FBS and gonadosomatic index (r = 0.428, P = 0.015).

The androgen receptors (AR) are the functional mediators of androgen hormones and specially testosterone in male gender. [28]. It is suggested that ARs are located on four major types of testicular cells and play a crucial role in the testis growth and development, production of testosterone from leydig cells, normal spermatogenesis, and germ cell growth [48]. Our data showed that T2DM decreased AR expression in testis, which is in agreement with Prabhu et al. study [34] that applied the immunostaining method. They suggested that testicular AR density in diabetic rats is remarkably lower than healthy ones. Via conducting Western blot analysis, we found that AR expression in the rats testis was increased after supplementation. Feeding the rats with *L. plantarum* for eight weeks did increase AR expression, compared to the control group (Fig. 3). Multiple studies have suggested that any decrease or elimination of ARs in testis can lead to an increased atrophy and cell apoptosis which causes smaller testis size and hypotestosteronaemia [8, 35, 48]. Interestingly, AR expression also showed a strong correlation with testosterone tissue levels and histologic parameters including tissue spermatid count, leydig cell number, and diameter of seminiferous tubules that could be referred as the impact of AR on testis development (Table 2) and kisspeptin expression (r = 0.943, P < 0.001).

It is well established that kisspeptin of the hypothalamus plays a pivotal role in releasing GnRH from hypothalamus ARC nucleons and triggering the HPG axis signaling [19]. Additionally, it is already known that kiss1 mRNA is expressed in several other peripheral tissues like testis. The testis releases the highest amounts of kisspeptin into the circulation [38]. In our study T2DM lead to diminished kisspeptin expression. Following supplementation with *L. plantarum* and inulin raised the level of testicular kisspeptin expression numerically; although the improvement reached the significant level only in the DI group (Fig. 3). Kisspeptin also showed a strong correlation with testosterone tissue levels and histologic parameters including tissue spermatid count, leydig cell number, and diameter of seminiferous tubules (Table 2). As far as we know, there is no study concerning the effects of T2DM and gut microbiota on testicular kisspeptin expression. Role of the testicular kisspeptin in endocrine control of the male fertility is under study [38, 47].

Salehi et al. [38] in agreement with Anjum et al. [4] suggested that testicular kisspeptin is expressed in leydig cells. These cells are the major source of testosterone production. Leydig cells produce testosterone in response to HPG axis signals, carried by luteinizing hormone (LH) from hypothalamus [19]. It is suggested that diabetes, obesity, and aging could diminish leydig cell count and function via inducing atrophy and oxidative stress. This defect brings about impaired spermatogenesis and semen volume [2, 5, 33]. In our study, the diabetic rats supplemented with *L. plantarum* and inulin or their combination had an increased leydig cell count. This can also be attributed to the antioxidant and anti-inflammatory effects of *L. plantarum* and inulin consumption, reported in earlier studies [29], Valenlia, [29]. Following the first chain of testis endocrine activity cascade, the increase in leydig cell number showed a strong positive correlation with testis testosterone level (r = 0.575, P = 0.002). However, serum testosterone levels did not change, possibly due to short study period. In confirmation  of our findings, Poutahidi et al. [33] studied testicular tissue of mice, consuming *L. rutei* and suggested that treated groups had increased leydig cell number and testis weight, compared to their oral counterparts. Probiotics supplementation also induced hyperplasia in rat testis which once was claimed to be achievable by administration of human gonadotropins like FSH and LH [14, 24, 25]. Probiotics also can induce anti-apoptotic effects [33]. Sefidgari et al. [39] suggested that administration of *L. plantarum* and inulin could indirectly decrease apoptosis in diabetic hearts via improving the metabolic status and reducing serum glucose and food intake. Directly, it could inhibit cardiac apoptosis through upregulation of antioxidants and Ob-R while downregulating expression of TNF-α in the cardiac tissue. Additionally, in our study, testis testosterone and leydig cell count showed a strong positive correlation with kisspeptin expression in rat testis (Table 2). This correlation may indicate that testicular kisspeptin might have an effect on the production of leydig cell testosterone, as well.

In the present study, diabetes caused a remarkable decrease in the diameter of seminiferous tubules along with decreased intratesticular spermatid count. Though supplementation with *L. plantarum* and inulin for 8 weeks was able to improve the diameter of seminiferous tubules as well as epididymal sperm qualitative parameters (Fig. 2). There were remarkable increases in the progressive motility and viability as well as decreased immotile sperms. Testicular spermatid count also increased. Although epididymal sperm concentration was numerically decreased after diabetes induction, this change was not statistically significant (Table 3). This consistency in testicular sperm production after STZ induction might refer to its probable compensatory mechanism in order to avoid any failure in the ultimate epididymal sperm concentration and/or short study period. In line with our results, several other studies found that gut microorganisms could promote reproductive ability in diabetic male rats [3, 10, 16]. In this regard, supplementation with *L. rutei* in rats improved seminiferous diameter as well as spermatogenesis [33]. These results were in agreement with the findings of Ham et al. [22] that suggested probiotics enriched with selenium could improve murine male fertility, fed a high-fat diet [28]. Probiotic translocation and consequently, the presence of the gut microbiota in the male urogenital tract could as well play a beneficial role in semen quality [10].

Our initial hypothesis based on previous studies was that combination of pro- and prebiotic as a symbiotic could show synergistic effects. In the present study, general outcome of treatment in DLI group significantly showed the expected beneficial effects, compared to control group. Although for some of the parameters, especially in terms of protein expression like testicular AR or kisspeptin expression, the impact of mere probiotic or prebiotic was significantly stronger. In fact, supplementation with *L. plantarum* or inulin offered stronger effects, whether statistical or numerical (Fig. 3). The authors would relate this point to the short duration of the intervention period. Possibly, proved synergistic and stronger effect of feeding symbiotic including impacts on gene or protein expression is achieved after a longer period. Hypothetically, it seems that direct treatment with probiotics and prebiotics will show their own therapeutic effects sooner than more strong effects of the concurrent administration. We know that the number of beneficial bacteria like Lactobacillus in diabetes is reduced. It is suggested that while prebiotics induce beneficial effects via secretion of gut peptides from enterocytes that contribute to reduction of diabetes complications independently, they change the microbial composition by gradually increasing the useful bacteria at a lower rate [43]. However, the science on the effects of microbiota on gene or protein expression in testis is in its infancy and due to the limited evidence in this field, it is difficult to justify the relevant mechanism. In addition, the significant correlation between histologic and sperm parameters and testicular protein expression in all groups is noticeable (Table 2). After all, more experimental studies in more optimized condition need to be conducted.

Finally, our results suggested that supplementation of *L. plantarum* and inulin in diabetic male rats for 8 weeks could have beneficial impacts on fertility outcomes including increased spermatid count and sperm motility and viability, improved leydig cell number and seminiferous tubule diameter as well as testicular AR and kisspeptin expression.

The novelty of our study was to investigate the fertility parameters in relation to testicular kisspeptin in diabetic rats fed either by probiotic or prebiotic or both. Also, we studied the expression of testicular kisspeptin which is a more valid method, compared to serum or tissue fluid levels. However, the study faced some limitations including obligatory shortened duration of the supplementation due to high rate of death in the groups not being supplemented. In addition, we could not assay hypothalamic kisspeptin in parallel with testicular levels to figure out a more detailed mechanism and correlation between these two tissues based on kisspeptin. LH was another basic fertility parameter that was better to be measured; however, we could not measure it due to deficit. More experiments on larger populations of rats in longer periods are suggested to be conducted. It is also proposed that fertility markers be examined simultaneously in serum, tissue, and gene level to obtain more comprehensive results.

## Conclusion

The present study showed the effectiveness of *L. plantarum* 1085 (ATCC 8014) and inulin supplementation on fertility parameters and reproductive ability of rats with T2DM. Our study also suggested the role of testicular kisspeptin and AR on testis development and function. Finally, it was concluded that separate or concurrent administration of *L. plantarum* and inulin can be considered a new approach in promoting fertility in T2DM.

## Data Availability

Data and materials are available by corresponding author whenever requested.

## Abbreviation

T2DM: Type 2 diabetes mellitus
*L. plantarum*: *Lactobacillus plantarum*
T: Testoestrone
GnRH: Gonadotropin releasing hormone
